# Non-infectious mimics of community-acquired pneumonia

**DOI:** 10.1186/s41479-016-0002-1

**Published:** 2016-04-12

**Authors:** Andrew D. Black

**Affiliations:** Wits Reproductive Health and HIV Institute, Faculty of Health Sciences, University of the Witwatersrand, Johannesburg, South Africa

**Keywords:** Non-infectious, pneumonia, differential diagnosis, community-acquired pneumonia, mimics, non-resolving

## Abstract

Community-acquired pneumonia (CAP) is a common cause of presentation to healthcare facilities. The diagnosis of CAP is usually made in patients with suggestive symptoms, signs, and radiological features. A number of non-infectious conditions, including neoplastic lesions, pulmonary oedema, pulmonary embolism, drug-induced pneumonitis, diffuse alveolar haemorrhage syndromes, cryptogenic organising pneumonia and acute eosinophilic pneumonia, may present in a similar way and mimic CAP. These other conditions are often only thought of after patients that are being treated as CAP fail to respond to therapy. The non-infectious mimics of CAP require early diagnosis and appropriate treatment to decrease patient morbidity and mortality. This article is intended to create an awareness of the non-infectious mimics of CAP and highlight some of the more frequent conditions as well as those that require early diagnosis and treatment to prevent a poor outcome.

## Background

Community-acquired pneumonia (CAP) is a frequent and treatable cause of persons presenting to healthcare practitioners. CAP is an acute infection of the pulmonary parenchyma in a person who has not had regular exposure to the healthcare system or a recent hospital admission [1].

CAP is considered as a diagnosis in patients presenting with the CAP syndrome, which comprises of two or more of the following symptoms or signs: fever, new or increased cough, sputum production, dyspnoea, pleuritic chest pain, confusion, crackles or signs of consolidation on chest auscultation, and a leukocytosis [2]. A chest radiograph with infiltrates compatible with acute pulmonary infection usually “clinches” the diagnosis of CAP in the attending clinician’s mind [3]. Despite advances in healthcare and antibiotic therapy, CAP remains a leading cause of mortality in South Africa [4].

Unfortunately, the symptoms and signs of the CAP syndrome are not specific and, even with chest radiograph features compatible with acute pulmonary inflammation, 5–17 % of patients admitted to hospital with CAP may actually have a non-infectious mimic of CAP [2, 5]. These figures are likely to be an under-representation of the incidence of mimics of CAP in clinical practice, as the published data on CAP usually only includes patients with a clear diagnosis of CAP. A further diagnostic challenge is that CAP and the mimics of CAP may coexist in a patient, such as pneumococcal pneumonia and tuberculosis, pulmonary oedema with secondary infection or a pulmonary embolism in a bed bound patient [6]. These non-infectious mimics of CAP (Table 1) require specific management and without appropriate treatment often have poor outcomes [7].

**Table 1:** Non-infectious mimics of community-acquired pneumonia

Cardiovascular	
Pulmonary oedema	
Pulmonary embolism	
Neoplastic	
Lung cancer	
Endobronchial metastases	
Lymphoma	
Immunological disorders	
Vasculitic diffuse alveolar haemorrhage	
Wegener’s granulomatosis	
Cryptogenic organising pneumonia	
Acute interstitial pneumonia	
Sarcoidosis	
Pulmonary alveolar proteinosis	
Systemic lupus erythematosus	
Polymyositis and dermatomyositis	
Acute and chronic eosinophilic pneumonia	
Drug toxicity	
Radiation pneumonitis	

Infectious biomarkers such as highly sensitive C-reactive protein (hCRP) and procalcitonin (PCT), while suggestive of an infectious aetiology, may be raised in many of the mimics of CAP and do not have sufficient sensitivity or specificity to rule in CAP or to rule out the non- infectious mimics; however, they can complement the diagnostic process [8]. Investigations to determine a causative agent and confirm the infectious aetiology of the CAP syndrome yield low results and are often not done in all patients with a diagnosis of CAP [9].

While there is a wide differential diagnosis for the CAP syndrome, the high incidence of CAP and the relatively low incidence of CAP mimics makes it appropriate to begin early guideline-directed empiric antibiotic therapy for CAP, as early antibiotics improves CAP outcomes [10]. Unless there is strong suspicion of a CAP mimic at the time of presentation due to atypical features such as multi-system disease or peripheral blood eosinophilia, the mimics of CAP are usually only thought of in patients with treatment failure for CAP.

Patients with CAP usually show improvement in tachypnoea, tachycardia, and fever within 72 h of starting antibiotic therapy. Chest radiograph abnormalities take up to 12 weeks to resolve but stabilisation and reduction of the infiltrates occurs earlier [11]. Treatment failure can be considered in three main groups: (i) progressive pneumonia, where patients show continued deterioration despite antibiotic treatment; (ii) slowly resolving pneumonia where there is a delay in clinical improvement beyond 72 h or failure to show chest radiograph improvement after 2 weeks after initiation of antibiotic treatment; (iii) non-responding pneumonia where pulmonary infiltrates persist on the chest radiograph beyond 12 weeks [7, 11].

In a patient with treatment failure, consideration needs to be given to known host factors that may increase the risk of failure such as advanced age, congestive cardiac failure, hepatic disease and alcohol abuse. The most common causes of treatment failure are infection with an antibiotic resistant or an unusual organism or an infectious complication of CAP such as an empyema [11]. The initial management in these patients would be a repeat chest radiograph to look for complications, an escalation of antibiotic therapy while reviewing any initial microbiological specimens, and sending further microbiological specimens looking for resistant or unusual organisms. In areas with high human immunodeficiency virus infection and tuberculosis prevalence, early testing for both conditions is strongly recommended in patients presenting with the CAP syndrome. A complete re-evaluation of the patient with a comprehensive history and examination with a full cardiac examination and cognisance of the non-infectious mimics of CAP needs to be conducted. Further investigations directed at excluding or confirming a mimic of CAP need to be considered. The urgency of further investigations is determined by the severity of the patient’s illness. A very high index of suspicion for an alternative diagnosis to CAP is needed in those patients who present with severe pneumonia in the absence of hypotension.

## Neoplastic lesions

Neoplastic lesions may mimic CAP in two ways; the most common is an endobronchial lesion, which may be a primary lung carcinoma or metastatic cancer (malignant melanoma, adenocarcinoma of the breast or gastrointestinal tract, or Kaposi’s sarcoma) [7]. Endobronchial lesions may cause obstruction leading to the accumulation of secretions and predispose the patient to distal infection with resultant fever. The obstructive lesion may not be apparent on the chest radiograph but hilar or mediastinal adenopathy, if present, should raise the index of suspicion. Computerised axial tomography (CAT) scan of the chest may show the obstructive lesion, and fibre-optic bronchoscopy with biopsy is indicated to establish a diagnosis, particularly in cases when sputum cytology is negative.

Certain malignancies may present with a pulmonary infiltrate and areas of consolidation without obstruction of a bronchus. The most common neoplasm to present in this way is the bronchioalveolar carcinoma (BAC), which is usually a mucinous adenocarcinoma of the lung [12]. Alveolar carcinoma is increasing in incidence and compared to other primary lung cancers occurs more frequently in women and non-smokers. Cough is usual and profuse bronchorrhoea, if present, should raise the index of suspicion, fever may be present but is unusual. The chest radiograph may show consolidation. Positron-emission tomography does not help differentiate between BAC and infectious pneumonia [13]. Diagnosis is made with sputum cytology or histology from specimens obtained from transbronchial biopsy. The prognosis of patients presenting with the pneumonic form of BAC is poor [13].

Both Hodgkin’s and Non-Hodgkin’s lymphoma can present with primary or secondary pulmonary involvement [7]. The usual radiographic appearance is nodules or masses, but a pattern suggestive of an infectious aetiology may occur. Loss of weight and/or hilar, mediastinal or peripheral adenopathy should prompt further investigation. Fibre-optic bronchoscopy and transbronchial biopsy may be diagnostic but usually a surgical biopsy is required to provide an adequate amount of tissue to confirm the diagnosis.

## Pulmonary oedema

Pulmonary oedema frequently presents as a CAP syndrome [2]. In patients with areas of altered pulmonary perfusion due to bullae, chronic obstructive pulmonary disease or valvular disease, pulmonary oedema may appear as a localised infiltrate on chest radiograph. An enlarged cardiac silhouette should increase the suspicion of cardiac disease. Primary cardiac disease with pulmonary oedema may predispose a patient to infectious pneumonia. Pneumonia may aggravate existing cardiac pathology such as cardiomyopathy, valvular disease or ischaemic heart disease, and induce or worsen congestive cardiac failure. The examination of a patient with respiratory symptoms should always include a thorough cardiac evaluation. In patients with CAP that deteriorate clinically despite antibiotic treatment, a new cardiac event such as an acute myocardial infarction or arrhythmia should be considered [14].

## Pulmonary embolism

Pulmonary embolism (PE) may present with the clinical and radiological features of the CAP syndrome. Early diagnosis and treatment of PE decreases mortality and the possibility of PE should be considered in patients presenting with the CAP syndrome. Biomarkers of infection such as hCRP and PCT, or of thrombosis such as highly sensitive D-dimers, may be raised in both PE and CAP and cannot be relied upon to differentiate between the two conditions; however, together with the clinical impression these biomarkers may assist in the decision to proceed with CT pulmonary angiography (CTPA) [15].

Patients should be risk stratified using a scoring system such as Wells’ criteria [16]. Patients with a low probability of PE with low D-dimer levels do not require CTPA [16]. Patients with a low probability of PE and high D-dimer levels (which may be due to infection) require independent clinical judgment with regards to further investigation for PE. Patients with a Wells’ score suggesting that a PE is likely, despite the possibility of CAP being an alternative diagnosis, do not require measurement of D-dimers and require further investigation for PE. In patients able to tolerate intravenous contrast media, CTPA is the investigation of choice. Where CTPA is contraindicated or not available, a ventilation perfusion (VQ) scan is an alternative; however, chest radiograph abnormalities may make interpretation difficult. Proximal vein lower-limb compressive ultrasound may diagnose a deep vein thrombosis in 36–45 % of patients with a PE [17]. Diagnosis of a deep vein thrombosis allows for full anticoagulation without confirming a PE with further imaging. However, the majority of patients with a proven PE will have a negative proximal vein lower-limb compressive ultrasound examination and will require further imaging.

## Drug-induced pneumonitis

A number of drugs, particularly cytotoxic agents, can cause pulmonary disease. Illicit drugs associated with pulmonary disease include heroin (smoked or injected) and crack cocaine. The use of illicit drugs should be included in a drug history. A comprehensive list of drugs that may cause pulmonary disease can be found at www.pneumotox.com. Fever, non-productive cough and dyspnoea are the usual presentation of drug-induced pneumonitis and occur within weeks or months of initiating therapy/use. Chest radiograph abnormalities may be absent early on but infiltrates usually develop and may localise to mimic pneumonia. A detailed drug history should be obtained from all patients at presentation. Some drugs such as methotrexate may predispose a patient to infection and are commonly used in conditions that may also be associated with pulmonary disease. High-resolution CAT scan of the chest and fibre-optic bronchoscopy are not helpful in the diagnosis of drug-induced pneumonitis, but may exclude the possibility of an infectious or other non-infectious aetiology as an alternative diagnosis.

## Diffuse alveolar haemorrhage syndromes

The diffuse alveolar haemorrhage syndromes are characterised by bleeding into the alveolar spaces and may result from a number of causes.

Haemoptysis is the usual predominant symptom but may be absent. General examination may reveal features of a vasculitic or rheumatological disorder. The chest radiograph often shows diffuse bilateral opacities, but a localised infiltrate may mimic pneumonia. Diagnosis is made with fibre-optic bronchoscopy and sequential bronchioalveolar lavage. Management is supportive and includes adequate oxygenation and correction of any coagulation abnormalities while trying to identify and treat the underlying cause [18].

## Cryptogenic organising pneumonia

Cryptogenic organising pneumonia (COP) is the idiopathic form of organising pneumonia and is classified under the idiopathic interstitial lung diseases. Patients are usually in their fifties or sixties and present with dyspnoea, cough and fever which may be of acute duration. The chest radiograph features of COP are typically bilateral with areas of patchy or diffuse consolidation or ground glass infiltrates. More focal infiltrates may resemble pneumonia. Management includes excluding known causes of COP such as drugs and the rheumatoid diseases. Diagnosis requires open lung biopsy. Treatment with glucocorticoids usually results in rapid improvement [19].

## Acute eosinophilic pneumonia

Acute eosinophilic pneumonia usually presents with an acute history of non-productive cough, dyspnoea, and fever. Diffuse parenchymal opacities, rather than localised consolidation, are typically seen on chest radiograph. Initial full blood count with a differential shows a neutrophilic leucocytosis but patients with severe progressive disease may develop an eosinophilia. Diagnosis is made by fibre-optic bronchoscopy with bronchioalveolar lavage demonstrating a significant eosinophilia. Without treatment, patients develop progressive respiratory failure. Treatment with glucocorticoids results in rapid improvement [20].

## Conclusion

As CAP is common, the majority of patients presenting with the CAP syndrome will have CAP and require the early administration of appropriate antibiotics as per regional guidelines. While any individual mimic of CAP may be uncommon, as a group they contribute to a significant proportion of patients admitted to hospital for the CAP syndrome. Initial evaluation of patients presenting with the CAP syndrome should include a detailed drug history and enquiry about risk factors for thromboembolic disease. Examination should include a search for extrapulmonary and in particular cardiac disease that may indicate a higher possibility of a non-infectious mimic of CAP being responsible for the pulmonary presentation.

Awareness and consideration of mimics of CAP, particularly in patients with treatment failure or clinical deterioration, should expedite further investigations. High-resolution chest CAT scan with mediastinal windows may assist in classifying the aetiology as infectious or non-infectious [21]. Early fibre-optic bronchoscopy with bronchioalveolar lavage and transbronchial biopsy should form part of the work up where a non-infectious mimic of CAP is suspected. Early correct diagnosis allows for appropriate therapy and decreased patient morbidity and mortality.

### Search strategy

Relevant articles were identified by searching PubMed and Google Scholar for articles (published in English and restricted to adults) including in their titles or abstracts the term “pneumonia” combined with the any of the following terms: “non-infectious”, “mimics”, “differential diagnosis”, “non-resolving”. More citations were identified from references in these initial searches.
